# Primordial Enemies: Fungal Pathogens in Thrips Societies

**DOI:** 10.1371/journal.pone.0049737

**Published:** 2012-11-21

**Authors:** Christine Turnbull, Peter D. Wilson, Stephen Hoggard, Michael Gillings, Chris Palmer, Shannon Smith, Doug Beattie, Sam Hussey, Adam Stow, Andrew Beattie

**Affiliations:** 1 Department of Biological Sciences, Macquarie University, Sydney, New South Wales, Australia; 2 Department of Natural Resources, Northern Territory Government, Alice Springs, Northern Territory, Australia; University of Osnabrueck, Germany

## Abstract

Microbial pathogens are ancient selective agents that have driven many aspects of multicellular evolution, including genetic, behavioural, chemical and immune defence systems. It appears that fungi specialised to attack insects were already present in the environments in which social insects first evolved and we hypothesise that if the early stages of social evolution required antifungal defences, then covariance between levels of sociality and antifungal defences might be evident in extant lineages, the defences becoming stronger with group size and increasing social organisation. Thus, we compared the activity of cuticular antifungal compounds in thrips species (Insecta: Thysanoptera) representing a gradient of increasing group size and sociality: solitary, communal, social and eusocial, against the entomopathogen *Cordyceps bassiana*. Solitary and communal species showed little or no activity. In contrast, the social and eusocial species killed this fungus, suggesting that the evolution of sociality has been accompanied by sharp increases in the effectiveness of antifungal compounds. The antiquity of fungal entomopathogens, demonstrated by fossil finds, coupled with the unequivocal response of thrips colonies to them shown here, suggests two new insights into the evolution of thrips sociality: First, traits that enabled nascent colonies to defend themselves against microbial pathogens should be added to those considered essential for social evolution. Second, limits to the strength of antimicrobials, through resource constraints or self-antibiosis, may have been overcome by increase in the *numbers* of individuals secreting them, thus driving increases in colony size. If this is the case for social thrips, then we may ask: did antimicrobial traits and microbes such as fungal entomopathogens play an integral part in the evolution of insect sociality in general?

## Introduction

Animal aggregations attract many kinds of enemies including predators and parasites, and this is especially the case for the social insects [1]–[3]. Microbial pathogens were ancient selective agents that influenced many fundamental aspects of multicellular evolution [4] and instigated, or at least maintained, major defence mechanisms such as sexual reproduction and immune systems [5]–[8]. With this in mind, we wondered if specialised fungal pathogens were among the most ancient enemies of social insects perhaps because they were common, pre-existing soil-dwelling species.

Insects in general have been battling microbial pathogens for at least 400 million years [9], [10]. Fossil evidence places most of the earliest social insects in the mid Cretaceous, about 100 million years ago (mya) [11], [12], although a fossil termite has been identified from early Cretaceous deposits [13]. Our study is prompted in part by the remarkable fossil fungal entomopathogen *Paleoophiocordyceps coccophagus* that has been identified from the early Cretaceous [14], showing the antiquity of fungal entomopathogens. While this particular species parasitised scale insects, the fungal genus is an early component of the lineage of *Ophiocordyceps*, known to have attacked ants 48 mya [15] and its derivative, the genus *Cordyceps*, is an important and widespread extant insect fungal entomopathogen [16]. *Cordyceps* was capable of major host shifts between distantly related host insects; one such shift dated at 43±13 mya [17] suggests that thrips may have been vulnerable. Selection by fungal entomopathogens appears to have been on-going as *Metarhizium (Cordyceps)* is recorded from more recent deposits dated at 7.2–8.2 mya [18]. Today, *Beauveria* (*Cordyceps*) *bassiana* occurs naturally with thrips [19] and is considered sufficiently lethal to be a biological control agent [20].

The particular vulnerability of social insects to microbial pathogens has been hypothesised for several decades [21], [22] and more recently the central role of antibiotics in non-hymenopteran social insects including termite evolution has been documented [23]. We have demonstrated increased levels of antimicrobial activity in species of bees and thrips selected to exemplify a gradient of social organization from solitary to semi-social to eusocial. Antimicrobial activity was dramatically higher in the most rudimentary semi-social species than in solitary species [24]–[26] which strongly suggests that selection by microbial pathogens affects even the most rudimentary societies. Overall, the literature on both Hymenopteran and Isopteran social evolution suggests that microbial pathogens have been present in the environments in which sociality first evolved and that they are important selective agents to this day. Further, if the early enemies of insect societies were microbial, then the continued assembly of social traits may have been possible only in the presence of strong antimicrobials.

In this context, we assayed the anti-fungal activity of 6 thrips species against the native entomopathogen *Cordyceps bassiana* according to group size and the degree and type of social organisation (solitary, communal, social and eusocial). Our hypothesis was that there is a correlation between anti-*Cordyceps* activity and group size and that *Cordyceps* inhibition, measured from equivalent numbers of individuals, would be greatest in species that formed colonies, whether social or eusocial.

## Materials and Methods

### (a) Study animals

The species examined in this study were selected in accordance with two criteria: first, level of organisation (solitary, communal, social, eusocial) and second, the availability of sufficient numbers to achieve serial dilution assay replicates for statistical comparison. All genera studied are members of the sub-Family Phlaeothripinae and *all* social and eusocial species in the Order Thysanoptera are placed in the single genus *Kladothrips* [27], [28]. Details are shown in Table 1. Thrips are common invertebrates and their use is unregulated, requiring no ethics approval.

**Table 1:** Characteristics of the experimental thrips species and details of number of replicates, gall numbers and contents.

Species (# replicates)	Level of Organisation	Biology. Number galls per replicate, 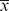 ± S.E.	Number individuals per gall or leaf curl, 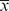 ± S.E.
*Kladothrips arotrum* (7)	**social**: female + offspring	gall-maker on *Acacia* 2.0±0.20	313±55
*K.antennatus* (5)	**social**: female + offspring	gall-maker on *Acacia* 3.2±0.21	141±31
*K.intermedius* (5)	**eusocial**: queen + soldiers + dispersers	gall-maker on *Acacia* 3.4±0.24	155±27
*Teuchothrips ater* (4)	**communal**	assemble in leaf curl, on *Pittosporum*	23±4.4
*Haplothrips froggatti* (6)	**solitary**	collected on *Cenchrus ciliaris*	
*H. varius* (6)	**solitary**	collected on *Ptilotus sp.*	

### (b) Antifungal concentrations

Living *Kladothrips arotrum, K. antennatus, K. intermedius, Teuchothrips ater, Haplothrips froggatti* and *H. varius thrips* (Table 1) were washed to obtain cuticular antifungal compounds using previously established methods [24]–[26], [29]. *K. arotrum* and *K. antennatus* are regarded technically as solitary by thysanopterists but are clearly social for the purposes of this research as there are hundreds of juveniles packed tightly into a gall. For all species, 400 individuals were pooled (Table 1) to produce each replicate which was then washed with 90% ethanol for 5 minutes, and rinsed three times for maximum extraction. These solutions were then dried in a rotary evaporator and the subsequent extract resuspended in a known volume of Luria Bertani broth (LB) to produce solutions containing a known concentration of thrips equivalents per µl. For each species and replicate, a gradient of antifungal extract concentration was created across a 96 well-plate so that there was a concentration of 200 thrips-equivalents in the first well that was reduced by half in each successive well [29]. Each well of the dilution series was inoculated with 100 µl of a standardized concentration of *Cordyceps bassiana* conidia. Controls for each well were: 1) equivalent volumes and concentrations of extract alone with 100 µl LB broth. This checked for any growth in the extract alone. 2) 100 µl of *C. bassiana* suspension with 100 µl LB broth which provided the normal growth curve for *C. bassiana*. Prepared plates were placed in a plate reader and recordings of optical density (OD) were made every 15 minutes for 48 hours using a wavelength of 405 nm at 25°C to compare the rates of germination of treated *C. bassiana* spores and subsequent hyphal growth. Controls for potential antimicrobial activity in the plant gall and leaf-curl tissue had been carried out previously and revealed no activity [26].

### (c) Analyses

We characterised the change in antifungal activity of each replicate of each species along the concentration gradient that permitted an assessment by inspection of differential patterns of change based on degree of sociality. We used the *lmList* function in the *nlme* package of version 2.14 of the *R* statistical environment [30] to fit linear models of Adjusted Density (defined as the difference between treatment and control OD) versus time grouped by species (6 levels) and concentration (6 levels). Simple regression for repeated measures over time is a valid technique for characterising linear trends over time [31]. Replicate runs for each species were pooled to perform the regressions (Table 1). The fitted slope for each species at each concentration characterised the behaviour of extracts over time as follows: a fitted slope greater than zero indicated the extract facilitated microbial growth at that concentration; a slope not significantly different from zero indicated the extract was no more effective than the control and finally, fitted slopes less than zero indicated the extract inhibited fungal growth relative to the control (two-tailed t-test, Table 2). This approach did not involve comparing species and did not require unplanned *post hoc* comparisons.

**Table 2:** Results of testing the significance of the slope of fitted linear models.

		Concentration (Thrips-equivalents)					
Species	N	200	100	50	25	12.5	6.25
*Haplothrips frogatti*	25	−2.21 (0.03)	−1.88 (0.06)	−1.30 (0.20)	−0.31 (0.75)	−0.67 (0.50)	1.93 (0.05)
*Haplothrips varius*	25	−2.48 (0.01)	−0.91 (0.06)	−1.50 (0.13)	−0.96 (0.34)	−0.26 (0.79)	0.92 (0.36)
*Kladothrips antennatus*	20	−11.52 (0.00)	−5.06 (0.00)	−2.85 (0.00)	−1.00 (0.32)	1.80 (0.07)	2.44 (0.02)
*Kladothrips arotrum*	10	−8.14 (0.00)	−6.84 (0.00)	−4.23 (0.00)	−1.73 (0.08)	−0.45 (0.66)	0.18 (0.85)
*Kladothrips intermedius*	20	−8.69 (0.00) *	−7.1 (0.00)	−3.75 (0.00)	−0.77 (0.44)	−0.54 (0.59)	0.18 (0.86)
*Teucothrips ater*	15	0.57 (0.57)	0.73 (0.47)	−1.2 (0.23)	−1.36 (0.18)	0.04 (0.97)	−0.34 (0.74)

* N = 15.

A two-tailed t-test was applied to test the null hypothesis that a given slope was equivalent to zero. A significance level of 0.05 was used to determine if the null hypothesis could be rejected. The values are the t-value and p-value reported by the lmList function of the R package lm rounded to two decimal places. N-values represent the number of data points used in each model fit. The t-test degrees of freedom are therefore N–2.

## Results

**Figure 1:**
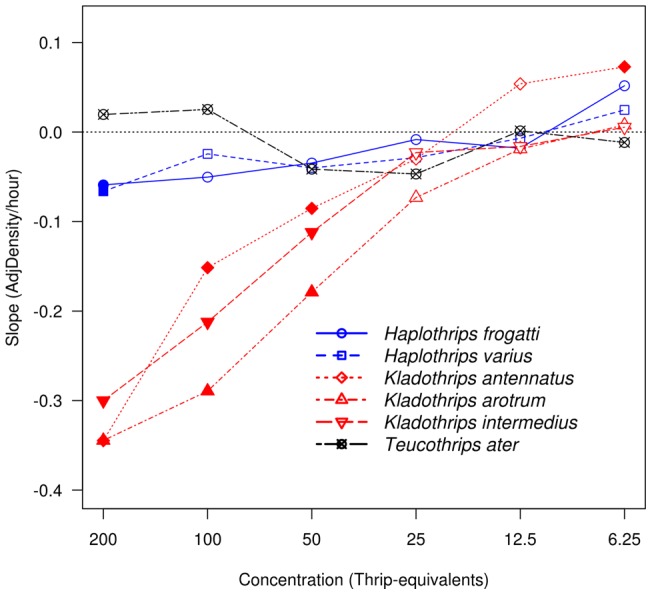
Antifungal activity in social/eusocial, communal and solitary thrips species. Slopes of fitted regressions of AdjDensity ( =  difference between treatment and control OD) on time, plotted against 6 concentrations of antifungal extract from 6 thrips species assayed against the entomopathogen *Cordyceps bassiana*. The species, their levels of organization and the number of replicates are given in Table 1. Red indicates social *Kladothrips arotrum* and *K. antennatus* and the eusocial *K. intermedius*; black indicates communal *Teucothrips ater* and blue indicates solitary *Haplothrips froggattii* and *H. varius.* Negative slopes indicate antifungal effect and magnitude indicates strength. Filled symbols indicate slopes significantly different to zero (Table 2).

The results are summarised by Fig. 1 and Table 2 which show strong evidence for inhibition of fungal activity in the social species: *Kladothrips antennatus*, *K. arotrum* and the eusocial species *K. intermedius* with highly significant negative slopes. In contrast, the solitary species *Haplothrips frogattii* and *H. varius* extracts showed no significant antifungal activity, with slopes close to zero, except at the highest concentration of 200 thrips-equivalents. The communal *Teucothrips ater* also showed no significant inhibitory activity and there was even a hint that at high concentrations the extract facilitated fungal growth (i.e. slope approximately 0 but intercept significantly >0). As this declined with decreasing concentration, regressions for this species were indistinguishable from controls. Fig. 1 reveals the quantitative nature of the ‘combat’ between the fungus and the antifungals as, at the lowest concentration, there appeared to be insufficient antifungal molecules to suppress fungal growth and that hyphae may have developed, as in the case of the single positive value for *K. antennatus* at 6.25 thrips equivalents.

## Discussion

Fig. 1 shows that antifungal activity was absent or very low in solitary and communal species but high and effective in social and eusocial species. The solitary species, *Haplothrips froggatii* and *H. varius* showed no activity except at the highest concentrations, suggesting that even solitary species, when crowded, might produce antifungal compounds. However, we do not know if they aggregate to this density in nature. There was a suggestion that extracts from the communal species, *Teucothrips ater*, showed a little antifungal activity in the range of 25–50 thrips-equivalents, perhaps approximating natural densities within curled leaves. However, at high concentrations, there was some evidence that their secretions promoted fungal growth. The social *Kladothrips arotrum* and *K. antennatus*, and the eusocial *K. intermedius* consistently exhibited stronger antifungal activity. As these and all other social and eusocial thrips species belong to the single genus *Kladothrips*, it might be argued that antifungal activity is somehow related to inhabiting *Acacia* galls rather than to social organisation. However, the pattern of antifungal activity being associated only with social and eusocial thrips species agrees with previous parallel research in bees [24], [25]. Further, we have also previously shown that gall tissue provided no antimicrobial activity [26] so that antifungal defence in these crowded conditions was derived from the insects. Finally, all activity ceased when the group size was 25 thrips equivalents or less, suggesting a threshold for the appearance of this type of defence. Microbial pathogens have shaped major evolutionary trends in defensive mechanisms in many kinds of animals including insects and vertebrates [32]–[34] and there is abundant evidence of their impact on social insect colonies, whether hymenopteran, isopteran or thysanopteran [23], [33]–[35]. Early insect lineages in which social traits were being assembled very likely encountered fungal entomopathogens [14], [35] that required frontline (i.e. non-immune) antifungal defences. These would prevent infection by killing spores or conidia before they germinated and penetrated the integument; prevention rather than cure. The importance of environmental factors in the origin and evolution of eusociality has recently been emphasised, but the role of fungal pathogens, although well known in the termites and ants [22], [23], has received less attention in this literature [36]. This study strongly supports the view that the shortlist of pre-adaptations (traits) generally considered essential to eusocial evolution, such as the potential for the division of labour, tunnelling behaviour, nest provisioning and guarding against intruders [36], should include, in addition, the ability to defend against pathogenic microbes.

Our data prompt some further thoughts on the role of fungal entomopathogens in social insect evolution: The propensity for generations to remain together, inherently increases group size and enhances social organisation, manifestly increasing the need for antifungal defence, as shown by the present data and other studies [21]–[23], [37]. This leads to the possibility that specialised fungal entomopathogens present in the geological epochs during which eusociality first evolved experienced the presence of increasingly stronger antifungal compounds, intensified as the degree of sociality and group size increased. Thus, the traits that enabled nascent colonies to combat microbial pathogens appear to have been fundamental to social evolution in thrips. In this context, selection by entomopathogens might have been instrumental in social and eusocial evolution being, in its early stages, driven by two possible defence strategies: the first an increase in the strength of antifungal compounds, and this has been demonstrated [24], [26]. However, limits to this response such as resource limitation or self-antibiosis [38], would require a second strategy such as increasing the *number* of individuals producing antifungal compounds. The efficacy of such an increase in colony size would have benefited from the appearance of the other, better-known, social traits. This scenario, for which our data provide some basic evidence, embeds a role for specialised insect pathogens in the early and subsequent evolution of thrips. The question arises as to whether or not antimicrobial defence traits and increases in the numbers of individuals secreting antimicrobials were both fundamental to social and eusocial evolution in the other great social insect groups.
